# Reducing routine laboratory tests in patients with isolated extremity fractures: a prospective safety and feasibility study in 246 patients

**DOI:** 10.1186/s13037-019-0203-7

**Published:** 2019-06-14

**Authors:** Raj. M. Amin, Alexander E. Loeb, Erik A. Hasenboehler, Adam S. Levin, Greg M. Osgood, Robert S. Sterling, Philip F. Stahel, Babar Shafiq

**Affiliations:** 1 Department of Orthopaedic Surgery, The Johns Hopkins Medical Institutions, 601 N Caroline Street, 5th Floor, Baltimore, MD 21287 USA; 2 Department of Specialty Medicine, Rocky Vista University College of Osteopathic Medicine, 777 Bannock St., Denver, CO 80204 Parker USA

**Keywords:** High-value care, Laboratory testing, Orthopaedic trauma surgery, Quality improvement

## Abstract

**Background:**

Daily routine laboratory testing is unnecessary in most admitted patients. The opportunity to reduce daily laboratory testing in orthopaedic trauma patients has not been previously investigated.

**Methods:**

A prospective observational study was performed based on a new laboratory testing reduction protocol for 12 months at two tertiary care trauma centers. Admitted patients with surgically treated isolated upper or lower extremity fractures were included (*n* = 246). The testing protocol consisted of a complete blood count (CBC) and basic metabolic panel (BMP) on postoperative day 2. Thereafter, tests were obtained at individual providers’ discretion. Patients were followed for 30 days postoperatively. The primary outcome was number of laboratory tests reduced. Secondary outcomes included provider protocol compliance, and adverse patient outcomes. Chi-squared tests were used to compare differences in categorical variables among the cohorts. Analysis of variance tests were used for continuous variables. The relative reductions in testing utilization were calculated using our division’s standard-of-care before program implementation (1 CBC and 1 BMP per patient per inpatient day). Significance was defined as *P* < 0.05.

**Results:**

Of the 246 patients, there were 45 protocol fall outs due to provider deviation (*n* = 24) or medically justified necessity for additional testing (*n* = 21). Across all groups, a total of 778 CBC or BMP tests were avoided, amounting to a 69% reduction in testing compared to the pre-implementation baseline. Ninety-five percent of protocol group patients were safely discharged either without laboratory testing or with one set of tests obtained on postoperative day 2. There were no 30-day readmissions or reported complications associated with the new laboratory testing protocol.

**Conclusions:**

In patients with surgically treated fractures about the elbow and knee, obtaining a single set of laboratory tests on postoperative day 2 is safe and efficacious in terms of reducing inappropriate resource utilization.

**Trial registration:**

retrospectively registered.

## Introduction

In 2017, United States health care expenditure accounted for nearly 18% of gross domestic product [1]. Resources are progressively strained with growing health care demands. Initiatives to reduce these costs are designed to decrease inappropriate resource utilization, including allogeneic red blood cell transfusions, urinary catheter use, continuous telemetry monitoring, and daily laboratory testing [2–7]. Daily laboratory testing has been recognized is one of the top 5 most overused diagnostic measures for hospitalized patients [2, 8, 9]. Often performed without high pretest probability, daily laboratory tests may yield abnormal results which provoke a cascade of interventions, as these results influence 60 to 70% of all medical decisions [10, 11]. Daily laboratory testing is also associated with patient harm, including technical errors, pain, and phlebotomy-associated anemia [12–14]. Reducing unneeded testing may therefore serve to improve patient care and satisfaction [15].

In nonoperative, clinically stable, hospitalized patients, reduction in laboratory testing has been implemented safely, proving daily laboratory testing to be unnecessary [16–20]. Data suggest that a 20 to 40% reduction in laboratory testing is safe, without increasing rates of death or hospital readmission [16]. Consequentially, internal medicine societies have adopted daily laboratory test reduction as a Choosing Wisely initiative in clinically stable patients [2].

However, research regarding the role of laboratory testing in the postoperative patient is limited, despite nearly 50% of nationwide hospital costs resulting from hospital stays for operative procedures [2, 21]. Prospective laboratory testing reduction initiatives have been implemented successfully in general, oncologic, and endocrine surgery [8, 22]. To our knowledge, a Choosing Wisely campaign that prospectively implements limited laboratory testing has not been performed in orthopaedic surgery inpatients or orthopaedic trauma inpatients.

Patients with isolated fractures requiring operative treatment commonly receive daily laboratory testing after surgery. Reasons for testing include diagnostic uncertainty, lack of feedback on test-ordering practices, provider team habit, or complex injury mechanisms [23, 24]. However, as orthopaedic trauma patient management has evolved, less perioperative laboratory tests may be needed, due to decreased perioperative complications with minimally-invasive surgical techniques, restrictive transfusion practices, and hospitalist co-management protocols [25–27].

Given the unclear benefit of daily postoperative testing in the orthopaedic trauma surgery population, we investigated the outcomes of reduced laboratory testing frequency in our patient population. We hypothesized that this initiative would result in a substantial reduction in laboratory test utilization without adverse clinical consequences.

## Methods

### Context

The orthopaedic trauma surgery divisions at 2 tertiary care academic hospitals (one Level 1 and one Level 2 trauma center) initiated this protocol. Patients with an isolated fracture were admitted to the orthopaedic trauma service; consultation with an internal or geriatric medicine specialist was available on an as-needed basis. Daily inpatient care, including treatment of medical comorbidities, was the responsibility of the orthopaedic surgery staff, with oversight by orthopaedic attending physicians. Before implementation of this protocol, patients admitted to the orthopaedic surgery service received daily laboratory testing, from day of admission until day of discharge. The following initiative was approved by our institutional review board.

### Intervention

During the 12-month study period (7/1/2017–6/30/2018), we implemented a prospective, restrictive laboratory testing protocol for orthopaedic trauma surgery patients. All operative patients admitted to the orthopaedic surgery service with isolated fractures at or below the elbow or knee were eligible for inclusion. This inclusion population represents a 65% proportion of our fracture-based orthopaedic care as chronicled in the 6 years prior to this study. Patients with femur or pelvis fractures were excluded given the elevated rate of perioperative medical complications and transfusions in this population compared to our inclusion population.

After surgery, providers were asked to order 1 complete blood count (CBC) and 1 basic metabolic panel (BMP) solely on postoperative day 2 for eligible patients. Providers retained the flexibility to order postoperative day 1 laboratory testing at their discretion if it was necessary for safe patient care. After postoperative day 2, providers were permitted to order laboratory tests only if the postoperative day 2 results or the patient’s condition indicated they were necessary. Patients discharged either on postoperative day 1 or the morning of postoperative day 2 before the scheduled lab draw did not undergo postoperative laboratory testing.

This initiative was designed according to previously established criteria for quality improvement interventions [28, 29]. It included the following: a project announcement with full support of the orthopaedic trauma surgery divisions and administration, a study coordinator group, unit-based patient care teams, and initiative education through peer learning. To assess protocol compliance and sustainability of the intervention, frequent individual provider feedback was given via e-mail in decreasing frequency over the intervention period. Provider feedback was given weekly in phase 1 (intervention months 1–4), given monthly in phase 2 (intervention months 5–8), and not given in phase 3 (intervention months 9–12).

### Study of intervention and measures

We collected data on preoperative patient characteristics, including age, sex, American Society of Anesthesiologists (ASA) score, and body mass index (BMI) value. We recorded whether fractures were open or closed, the anatomic location of the injury, and the treatment method. We recorded the following postoperative outcomes: number and timing of CBCs and BMPs ordered, duration of inpatient stay, allogeneic red blood cell transfusion status, in-hospital morbidity events, in-hospital deaths, and unplanned 30-day readmission to the same hospital system for any cause [8]. Providers who ordered laboratory testing against protocol were queried to determine whether testing was ordered in error or because of medical necessity. We analyzed all complications, including unplanned readmission, to determine the cause and whether reduced laboratory testing contributed to or caused the complication. Complication adjudicators were not blinded to protocol compliance status.

### Statistical analysis

Patients were assigned to 1 of the following 3 groups before analysis: 1) protocol group; 2) non-protocol group because of provider error (herein, provider-error group); or 3) non-protocol group because of medical necessity (herein, medical-necessity group). Chi-squared tests were used to compare differences in categorical variables among the cohorts. Analysis of variance tests were used for continuous variables. The relative reductions in testing utilization were calculated using our division’s standard-of-care before program implementation (1 CBC and 1 BMP per patient per inpatient day). Significance was defined as *P* < 0.05. All analyses were performed using JMP, version 12.1.0, software (SAS Institute, Cary, NC).

## Results

### Patient characteristics

Patient characteristics and fracture data are presented in Table 1. During the 12-month study period, 246 patients met inclusion criteria, of whom 201 (82%) received their first set of postoperative laboratory tests on postoperative day 2 (protocol group). Of the 45 (18%) patients who had postoperative day 1 laboratory assessment, 24 were because of provider error and 21 were because of medical necessity.

**Table 1:** Baseline characteristics of 246 orthopaedic trauma patients eligible for a limited laboratory assessment protocol

Parameter	All, n (%) (*n* = 246)	Protocol Group, n (%) (*n* = 201)	Non-Protocol Group, n (%)		*P* Value
			Provider Error (*n* = 24)	Medically Excluded (*n* = 21)	
*Baseline demographic data*					
Age, yr	47 ± 18^a^	46 ± 18^a^	42 ± 16^a^	63 ± 16^a^	0.0001
Female sex	117 (48)	97 (48)	6 (25)	13 (62)	0.04
ASA Score^b^	2.2 ± 0.6^a^	2.1 ± 0.6^a^	2.0 ± 0.7^a^	2.8 ± 0.5^a^	< 0.0001
BMI, kg/m^2^	29 ± 7.9^a^	28 ± 7.6^a^	28 ± 6.6^a^	32 ± 12^a^	0.15
*Injury characteristics*					
Open injury	38 (15)	32 (16)	4 (17)	2 (9.5)	0.73
Fracture location					0.21
Humerus	16 (6.5)	14 (7.0)	1 (4.2)	1 (4.8)	
Ulna/radius	36 (15)	28 (14)	3 (13)	5 (24)	
Patella	12 (4.9)	9 (4.5)	3 (13)	0 (0)	
Tibia					
Plateau	34 (14)	24 (12)	5 (21)	5 (24)	
Shaft	49 (20)	41 (20)	5 (21)	3 (14)	
Pilon	25 (10)	21 (10)	2 (8.3)	2 (9.5)	
Ankle	50 (20)	43 (21)	2 (8.3)	5 (24)	
Foot	24 (9.8)	21 (10)	3 (13)	0 (0)	
*Fracture treatment modality*					
ORIF	159 (65)	128 (64)	17 (71)	14 (67)	0.21
Intramedullary nail	40 (16)	33 (16)	5 (21)	2 (9.5)	
External fixation	31 (13)	27 (13)	1 (4.2)	3 (14)	
Arthroplasty	5 (2.0)	2 (1.0)	1 (4.2)	2 (9.5)	
Splint application	7 (2.8)	7 (3.5)	0 (0)	0 (0)	
Removal of instrumentation	4 (1.6)	4 (2.0)	0 (0)	0 (0)	

Abbreviations: *ASA* American Society of Anesthesiologists, *BMI* body mass index, *ORIF* open reduction and internal fixation

^a^Data presented as mean ± standard deviation

^b^ASA Score: A global assessment of a patient’s physical status made by the anesthesiologist before surgery. Ratings are from ASA I to ASA VI, where I is normal, II has mild systemic disease, III has severe systemic disease, IV has severe systemic disease that is a constant threat to life, V is a moribund patient not expected to survive the operation, and VI is a brain-dead patient awaiting organ harvest

Compared with the protocol group and the provider-error group, patients in the medical-necessity group were older (mean ± standard deviation: 63 ± 16 years, medical-necessity group vs. 46 ± 18 years, protocol group and 42 ± 16 years, provider-error group; *P* = 0.0001) and had a higher mean ASA score (2.8 ± 0.5, medical-necessity group vs. 2.1 ± 0.6, protocol group and 2.0 ± 0.7, provider-error group; *P* < 0.0001). There were no significant differences among the 3 groups in fracture location or open injury status (Table 1).

Fifteen percent of all patients had open fractures and 79% had lower extremity fractures (Table 1). Forty-four percent of patients were treated for tibial fractures, including tibial plateau, shaft, or pilon-variant fracture. Sixty-five percent of patients were treated with open reduction and internal fixation, 16% with intramedullary nailing, and 13% with external fixation.

### Perioperative outcomes

**Fig. 1:**
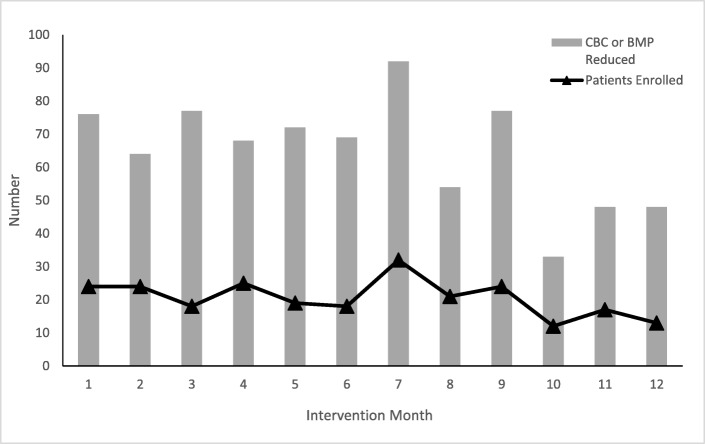
Perioperative laboratory test reduction and patient enrollment per month (CBC, complete blood count; BMP, basic metabolic panel)

The 246 patients included in our study accounted for 558 inpatient days. The mean number of CBCs and BMPs ordered per day was 0.27 ± 0.35. A total of 169 laboratory test blood draws were ordered. There were 390 laboratory test–free days, which equated to 70% of inpatient days and the avoidance of 778 CBCs and BMPs (Fig. 1).

The 201 patients in the protocol group accounted for 448 postoperative inpatient days and 92 postoperative laboratory test blood draws. Two patients in this cohort received postoperative transfusions. One patient underwent transfusion because of symptomatic anemia confirmed by postoperative day 2 laboratory testing (hemoglobin 6.9 g/dL). The lack of postoperative day 1 laboratory assessment did not delay the time to transfusion, as the patient was previously asymptomatic. The second patient underwent transfusion for symptomatic anemia on postoperative day 4 (hemoglobin 6.7 g/dL). Neither patient’s time-to-disposition was influenced because of reduced laboratory ordering practices as other unrelated factors (i.e. pain control) limited their discharge. Twenty-two laboratory test blood draws were obtained after postoperative day 2 on the basis of clinical appearance or initial laboratory test results. The 24 patients in the provider-error group accounted for 52 inpatient days and received 37 laboratory test blood draws.

Perioperative outcomes are shown in Table 2. In the entire cohort, there were seven 30-day all-cause complications not requiring readmission. Six patients had superficial surgical site infections, five of which were treated successfully with antibiotics, and 1 which required local wound care. One patient had a postoperative pulmonary embolism during the hospital stay. Six patients required unexpected 30-day readmission unrelated to our initiative: 1 postoperative pulmonary embolism (protocol group), 1 evaluation of previously undocumented heart murmur (protocol group), 1 return for dialysis (medical-necessity group), 2 internal fixation-related complications (1 protocol group, 1 medical-necessity group), and 1 admission for hyperkalemia (protocol group). The latter patient, who had a history of renal transplantation, had normal lab values throughout admission, including a normal CBC and BMP obtained on the day of discharge.

**Table 2:** Perioperative outcomes of 246 orthopaedic trauma patients eligible for a limited laboratory assessment protocol

Outcomes	All, n (%) (*n* = 246)	Protocol Group, n (%) (*n* = 201)	Non-Protocol Group, n (%)		*P* Value
			Provider Error (*n* = 24)	Medically Excluded (*n* = 21)	
RBC transfusion	2 (0.8)	2 (1.0)	0 (0)	0 (0)	–
LOS	2.3 ± 1.6^a^	2.3 ± 1.6^a^	2.2 ± 1.5^a^	2.9 ± 2.0^a^	0.251
30-day complication not requiring readmission	7 (2.8)	6 (3.0)	1 (4.0)	0 (0)	0.513
30-day unexpected readmission	6 (2.4)	4 (2.0)	0 (0)	2 (9.5)	0.135
CBC or BMP saved	778 (69)	712 (80)	31 (29)	35 (29)	–

Abbreviations: *BMP* basic metabolic panel, *CBC* complete blood count, *RBC* red blood cell, *LOS* length of hospital stay

^a^Data presented as mean ± standard deviation

A subgroup analysis was performed on the protocol group (Table 3). Sixty-two percent of protocol group inpatients were discharged before the scheduled postoperative day 2 lab draw. Though providers were allowed to order laboratory tests at their discretion after postoperative day 2, only 5.5% of protocol group patients received laboratory testing after postoperative day 2. For patients with hospital lengths of stay longer than 3 days, 79% did not require additional laboratory testing. Ninety-five percent of protocol group patients were safely discharged either without laboratory testing or with only one set of postoperative day 2 tests.

**Table 3:** Laboratory test usage breakdown for protocol group patients

Test Usage	Protocol Group, n (%) (*n* = 201)
No labs drawn^a^	124 (62)
Labs POD 2 only	66 (33)
Additional labs drawn after POD 2	11 (5.5)

Abbreviations: *POD* postoperative day

^a^No labs were drawn in the event of a hospital stay less than 3 days, as the first postoperative draw would have occurred on postoperative day 2

### Protocol adherence and intervention sustainability

**Fig. 2:**
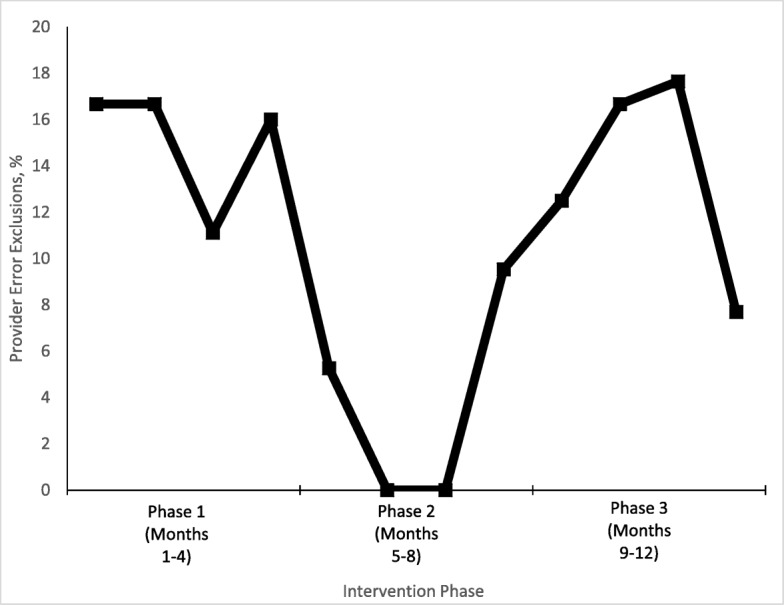
Percentage of provider errors by feedback phase in 246 orthopaedic trauma patients eligible for limited laboratory testing protocol

The percentage of patients who were tested because of provider error was 10% during the 12-month intervention (Fig. 2). By phase, the provider error rate was as follows: 15% during phase 1 (weekly provider feedback), 3.7% during phase 2 (monthly provider feedback), and 14% during phase 3 (no provider feedback).

## Discussion

Given increased strain on healthcare resources, there is a strong need to engage physicians in programs to reduce resource utilization while maintaining quality of care. To our knowledge, our initiative is the first to show that a prospective, restrictive laboratory testing protocol in selected orthopaedic trauma surgery patients decreases the number of inpatient laboratory tests ordered, while resulting in no identifiable 30-day adverse events related directly to reduced laboratory testing.

Although daily laboratory testing often has been part of routine postoperative inpatient orthopaedic care, little evidence exists to suggest this practice is necessary [30]. Common indications for inpatient hospitalization after orthopaedic surgery include pain control, mobility assistance, and disposition needs, none of which requires daily laboratory testing.

Our intervention suggests that reducing postoperative laboratory assessments in this population is safe. Compared with national 30-day unexpected readmission rates of 4 to 5% in this population, 2.4% of our patients had unexpected readmission [31]. After a thorough review of the aforementioned postoperative complications, we believe that none of the complications or unexpected readmissions was related to a lack of daily laboratory testing.

Previous laboratory testing reduction initiatives have been driven primarily by the field of internal medicine [32]. Iams et al. [8] showed that a multifaceted approach to CBC and BMP reduction in a large academic medical center yielded substantial 2-year declines in test utilization. Similarly, displaying costs associated with common tests and frequent appropriate care reminders have reduced the number of laboratory tests ordered [33, 34].

Research into reducing daily laboratory testing is less robust in the postoperative setting than in internal medicine. Iams et al. [8] aimed to reduce the number of CBCs and BMPs ordered for general surgery patients. In their study, general surgery providers decreased the number of daily CBCs, but there was no difference in the number of BMPs. This disparity may reflect differences in practice patterns between medical and surgical physicians and greater difficulty in changing the ordering habits of surgical providers.

Han et al. evaluated the effect of a financial incentive for neurosurgical staff aimed at reducing unneeded electrolyte testing [35]. Through financial incentives, their program realized a 47% reduction in unnecessary testing. However, their patients were comanaged by a hospitalist service; thus, their outcomes may not reflect surgical provider comfort with laboratory testing reduction. Morbidity that may have occurred as a result of laboratory testing reduction was not assessed.

Little evidence regarding deliberate laboratory testing reduction efforts exists in the field of orthopaedic surgery. A retrospective study found that, in patients who underwent anterior cervical discectomy and fusion, postoperative CBCs and BMPs prompted only an 0.89% rate of intervention and were therefore of minimal clinical utility [30]. We know of no studies in the field of orthopaedic trauma surgery that have examined the utility of daily laboratory testing. Also, to our knowledge, no study has sought to determine whether prospective restriction of laboratory testing is safe or whether providers would adhere to a reduced-testing protocol. This lack of data is detrimental: orthopaedic trauma to the distal lower extremity is the fifteenth most common reason for inpatient surgery in the United States (accounting for 79% of this cohort) [21].

In this study, our initiative reduced the utilization of CBC and BMP laboratory tests by 69%. These reductions in consumption and cost are critical because 33 to 50% of orthopaedic trauma patients are underinsured or uninsured [36, 37]. Potential savings also include activity-based opportunity costs, such as provider time invested in ordering and interpreting laboratory tests, as well as phlebotomist fees and supply costs. Unmeasurable costs were also likely saved by avoiding spurious false-positive results, which may have prompted invasive testing and specialist consultation.

Medical inpatient data suggest that phlebotomy-associated blood loss accounts for 20% of new-onset anemia in hospitalized patients [38]. In patients who are hospitalized for more than 10 days, a hemoglobin decrease of nearly 1 g/dL may be attributed to phlebotomy [38]. According to the reduction in laboratory testing achieved in our study, an estimated 2.2 to 3.9 L of whole blood loss was avoided in our 246 patients [39]. The effect of phlebotomy-associated anemia is reported primarily in medical patients, as opposed to surgical patients. This may be of more consequence for orthopaedic trauma surgery patients who experience phlebotomy-associated, surgery-associated, and injury-associated blood loss. Reducing the number of laboratory test blood draws may reduce the need for transfusions in these patients.

Our study also shows that residents physicians can successfully implement value-based care initiatives (Fig. 1). Previous studies on methods of improving provider compliance in Choosing Wisely efforts reported that combined approaches involving education, feedback, and knowledge of laboratory costs were critical to successful implementation [8, 15, 33, 35, 40, 41]. Our use of these methods among inpatient providers was associated with effective behavior change, as shown in the lower rates of physician error when feedback was used. We attribute this improved protocol adherence to a shared accountability model, a unit-based team concept, and a clinician-led effort [42]. Weekly feedback in phase 1 was associated with a moderate rate of provider error, likely due to the new introduction of the protocol. Monthly feedback in phase 2 was associated with lowest rate of provider error, as feedback was consistent and the new protocol was adopted. Zero feedback in phase 3 was associated with return to a moderate rate of provider error, likely due to protocol attrition.

Our reduced daily laboratory testing initiative shows several essential outcomes. The cost savings related to laboratory tests avoided during our study totaled $38,484; however, as discussed previously, multiple unmeasurable overhead costs, activity-related costs, and opportunity costs are not included in this amount. Considering a CDC estimate of 6.8 billion laboratory tests performed annually, the potential scalable benefit of our initiative is quite impactful [43]. The ability of inpatient providers to adhere to perioperative laboratory reduction protocols is not well established. Data suggest that less experienced physicians deliver lower-value care compared with more experienced physicians, and initiatives to reduce laboratory testing that have relied on surgeon compliance have been less successful than those involving non-surgical physicians [8]. Moreover, 8 of the 15 most common surgical procedures requiring hospitalization involve the musculoskeletal system [21]. Restrictive laboratory protocols in medical specialties involved in the perioperative care of orthopaedic trauma surgery patients may be another pathway where increased healthcare value may be obtained [21, 44]. Our study’s feedback-associated improvements in protocol adherence and enrollment show an increased participation in value-based medicine within our department and may present further opportunities for perioperative care improvement.

We acknowledge several study limitations. Our study population was relatively small. By including only patients admitted to the orthopaedic trauma surgery service, all operative general trauma surgery injuries were excluded. However, we anticipated that the inclusion of such nonoperative general trauma injuries would not change management, as a low rate of missed injury in general blunt trauma patients has been shown to not change initial nonoperative treatment [45]. While the rate of missed intraabdominal or vascular injury has not been established in the isolated distal extremity fracture patient, we presumed it to be low. Also, not all orthopaedic trauma service patients were included: pelvic or proximal extremity fracture patients were excluded due to a historical increased rate of acute transfusion [46]. Our readmission data only accounts for readmission within our healthcare system; however, regional referral practices typically involve contact with the patient’s index surgeon, or, when necessary, transfer of a patient with postoperative complications to the original surgical facility. Finally, although we report baseline utilization rates for laboratory tests, we are unable to compare pre- and post-intervention all-cause morbidity rates within the limitations of our institutional review board approval. However, we mitigated this limitation by using a broad definition of 30-day complications and performing a comprehensive review of each complication. We observed no complications related directly to laboratory testing reduction, nor any complications that should have changed inpatient treatment. Therefore, the use of pre-intervention comparison population would be unlikely to provide additional useful information regarding the safety of this initiative.

## Conclusions

Daily laboratory testing after orthopaedic trauma surgery is not always necessary. Reduction of daily laboratory testing in select orthopaedic trauma surgery patients decreases hospital resource utilization without attributable 30-day complications. In patients suitable for admission to an orthopaedic surgery service for isolated distal extremity fracture, restricting laboratory tests to one blood draw on postoperative day 2 is safe. Our model suggests that surgical subspecialties (including non-trauma orthopaedic subspecialties) may also be able to reduce the need for daily laboratory testing in their patients.

## Data Availability

The datasets generated and/or analysed during the current study are not publicly available due to institutional review board limitations but are available from the corresponding author on reasonable request.

## Abbreviations

ASA: American Society of Anesthesiologists
BMI: Body mass index
BMP: Basic metabolic panel
CBC: Complete blood count
LOS: Length of stay
ORIF: Open reduction internal fixation
POD: Postoperative day
